# Crescendo Transient Ischemic Attacks Due to Basilar Coccidioidal Meningitis With Coccidioma

**DOI:** 10.1177/2324709618813178

**Published:** 2018-11-19

**Authors:** Carlos D’Assumpcao, Arash Heidari, Katayoun Sabetian, Royce H. Johnson

**Affiliations:** 1 Kern Medical—UCLA, Bakersfield, CA, USA; 2 Ross University, Miramar, FL, USA; 3 Valley Fever Institute, Bakersfield, CA, USA

**Keywords:** coccidioidomycosis, coccidioidal meningitis, crescendo transient ischemic attack

## Abstract

Coccidioidal meningitis typically presents with symptoms that may include headache, altered mental status including personality changes, fever, nausea, vomiting, gait abnormalities, and focal neurological deficits. This is a case of coccidioidal meningitis that initially presented as 4 consecutive crescendo cerebrovascular transient ischemic attacks with focal neurological deficits that resolved within minutes. Imaging showed a left basilar coccidioma. Follow-up at 4 months showed treatment response to conservative therapy of fluconazole 1000 mg with a dexamethasone taper. Crescendo cerebrovascular transient ischemic attacks are a unique initial presentation of coccidioidal meningitis.

## Introduction

*Coccidioides* is found as 2 species, *immitis* and *posadasii*, that are clinically indistinguishable. Coccidioidomycosis is most commonly an asymptomatic infection. When symptomatic, it is commonly a pneumonia often mistaken for community-acquired pneumonia.^1^ However, in the few the disease can disseminate anywhere in the body. Meningitis is the most feared form of disseminated coccidioidomycosis. The most common presenting symptom is headache. Other symptoms include altered mental status, with or without fever, personality changes, nausea, vomiting, meningismus, gait abnormalities, and focal neurological deficits.^2^ Presented here is a case of coccidioidal meningitis that initially presented as multiple consecutive crescendo cerebrovascular transient ischemic attacks (TIAs).

## Case

A 64-year-old Hispanic male with diagnosis of pulmonary coccidioidomycosis 2 years prior at another institution and placed on therapy with 400 mg fluconazole daily for 1½ years. Initial serum coccidioidal immunodiffusion of IgM (immunoglobulin) and IgG were weakly reactive with complement fixation titers of 1:4. Symptoms resolved, and his physician decreased fluconazole to 200 mg daily for 4 months. He did well for 1 month until he developed left-sided headaches. After 2 weeks, he had 2 episodes of left arm and leg weakness without ability to walk and lower right facial palsy over a period of 10 minutes.

**Figure 1.**
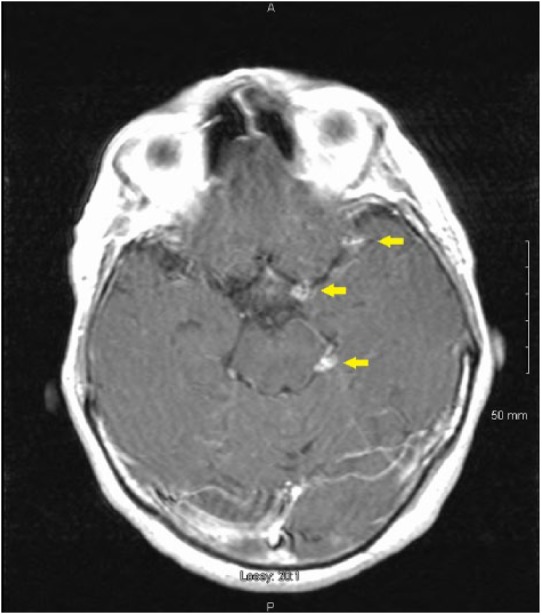
T1-weighted magnetic resonance image with gadopentetate dimeglumine contrast showing several nodular enhancements in the left peripontine area suspicious for coccidioma (arrows) in patient with multiple consecutive crescendo cerebrovascular transient ischemic attacks.

In the emergency department, while having his vitals taken, the patient had another episode of lower right facial palsy and left-sided weakness that resolved in 5 minutes. Computed tomography scan of brain without contrast as well as computed tomography angiogram of head and neck were completed and were unremarkable. Three hours later, the patient had another episode of right facial droop and left-sided weakness, followed by new-onset slurring of speech, resolving in 5 minutes. Magnetic resonance imaging of the brain showed no infarcts or intracranial hemorrhage, but it did show increased peripontine enhancement with several nodular enhancements in the basilar area suspicious for coccidioma (Figure 1). Lumbar puncture demonstrated opening pressure of 140 mm H_2_O, white blood cells 240 (34% lymphocytes, 39% monocytes, 18% neutrophils, 4% eosinophils, and 5% basophils), elevated protein 127 mg/dL (normal = 14-45 mg/dL), glucose 38 mg/dL (normal = 40-75 mg/dL), and coccidioidal compliment fixation titer of 1:4 diagnostic of coccidioidal meningitis. Serum coccidioidal immunodiffusion IgM and IgG were reactive with a compliment fixation titer of 1:16 (Table 1). He had a total of 4 cerebrovascular TIAs that were increasing in intensity and symptomology. He was placed on fluconazole 1000 mg daily^1^ and a dexamethasone 20 mg daily for 7 days then tapered by 4 mg every 4 days.^3^ He was discharged to be followed in clinic.

**Table 1.** Laboratory Results Summary. Serum serology at 2-month postdischarge were from a hospital visit for skin and soft tissue infection of left ankle unrelated to coccidioidomycosis.

Laboratory Test	2 Years Prior	Presenting Studies	2-Month Hospital Visit	4-Month Follow-up	Normal Range
CSF WBC		240 cells/cm^2^		50 cells/cm^2^	<5 cells/cm^2^
CSF protein		127 mg/dL		66 mg/dL	15-45 mg/dL
CSF glucose		38 mg/dL		43 mg/dL	40-75 mg/dL
CSF CF		1:4		<1:1	<1:1
Serum IgM	Weakly reactive	Very weakly reactive	Not reactive	Not reactive	Not reactive
Serum IgG	Weakly reactive	Reactive	Reactive	Reactive	Not reactive
Serum CF	1:4	1:16	1:4	1:8	<1:1

Abbreviations: CSF, cerebrospinal fluid; WBC, white blood cell; CF, compliment immunofixation titer; IgM/IgG, immunoglobulin immunodiffusion serology.

He had been and continues to be compliant with fluconazole therapy. At 2-month hospital visit for skin and soft tissue infection of left ankle unrelated to coccidioidomycosis, serum coccidioidal fixation titers were improved to 1:4. At 4-month follow-up, the patient had been asymptomatic. Lumbar puncture in office demonstrated white blood cell count of 50 (81% lymphocytes, 16% monocytes, 1% neutrophils, 1% eosinophils, and 1% basophils), protein 66 mg/dL (normal = 15-45 mg/dL), glucose 43 (normal = 40-75 mg/dL), and coccidioidal fixation titer of less than 1:1. However, serum coccidioidal fixation titers were 1:8 (Table 1).

## Discussion

To our knowledge, this is the first reported case of multiple consecutive cerebrovascular TIAs that were increasing in intensity and symptomology with each attack as the presenting manifestation of coccidioidal meningitis.^4^ Presenting characteristics are recurrent episodes of sudden discrete neurological symptoms that completely resolve within 24 hours that repeat with increasing duration, frequency, and severity indicative of critical narrowing of the involved artery.^5^ This patient had 3 coccidiomas in the basilar meninges (Figure 1). Four episodes of transient neurological deficits were documented. The coccidioidal meningitis with associated transient vasculitis was treated conservatively with fluconazole 1000 mg^1^ and a dexamethasone taper.^3^ Cerebrospinal fluid studies at 4 months showed treatment response. However, repeat serum serology at 2 and 4 months demonstrated persistent coccidioidomycosis (Table 1). Whenever logistically feasible, follow-up sooner than 4 months for CSF studies is ideal. There were no early indications of risk to dissemination at time of pulmonary coccidioidomycosis diagnosis. Physicians in areas endemic for coccidioidomycosis should be aware that cerebrovascular TIAs can be the initial presentation of coccidioidal meningitis.
